# Kaolin-supported Ag-Cu/g-C_3_N_4_ nanocomposite for efficient photocatalytic removal of methylene blue dye from aqueous solution

**DOI:** 10.1186/s11671-026-05135-y

**Published:** 2026-11-11

**Authors:** Azanaw Girmaw Mengstu, Bewketu Mehari, Minaleshewa Atlabachew, Abrham Biresaw Gebrye, Itziar Ruisanchez

**Affiliations:** 1 Department of Chemistry, College of Natural and Computational Sciences, University of Gondar, P.O.Box 196, Gondar, Ethiopia; 2 Chemistry Department, College of Natural and Computational Sciences, Debre Tabor University, Debre Tabor, Ethiopia; 3 Chemistry Department, College of Science, Bahir Dar University, Bahir Dar, Ethiopia; 4 Department of Chemistry, College of Natural Science, Injibara University, Injibara, Ethiopia; 5 Faculty of Chemistry, Department of Analytical and Organic Chemistry, Universitat Rovira i Virgili, Tarragona, Spain

**Keywords:** Kaolin, Ag-Cu/g-C_3_N_4_/kaolin Nanocomposite, Photocatalytic removal, Sunlight irradiation, Water treatment

## Abstract

Sunlight-driven photocatalytic degradation is a cost-effective and eco-friendly technology for removing organic pollutants from contaminated water. In our study, we use cheap and locally available kaolin as support for the fabrication of the 3-Ag-Cu/g-C_3_N_4_ nanocomposite via a simple chemical reduction method. The as-synthesized nanocomposite was characterized by Fourier transform infrared spectroscopy (FT-IR), X-ray diffraction (XRD), scanning electron microscopy (SEM), high-resolution transmission electron microscopy (HRTEM), energy-dispersive X-ray spectroscopy (EDX), and X-ray photoelectron spectroscopy (XPS), and all these techniques confirm the formation of the Ag-Cu/g-C_3_N_4_/kaolin nanocomposite. The photocatalytic activity of the nanocomposite was examined by analyzing its degradation efficiency for methylene blue (MB) dye under sunlight irradiation. The influence of different experimental parameters such as catalyst dosage, initial dye concentration, and solution pH on the photocatalytic degradation of MB was investigated. The 3-Ag–Cu/g-C₃N₄/kaolin NC exhibited a degradation efficiency of 98.95% under the optimized conditions after 120 min of sunlight irradiation. Scavenger experiments showed that the degradation of MB on 3-Ag–Cu/g-C₃N₄/kaolin NC was mainly controlled by the synergistic effects of photogenerated holes (h⁺) and superoxide radicals (•O₂⁻), with hydroxyl radicals (•OH) playing a relatively small role.

Clinical trial number: Not applicable

## Introduction

As the demand for colorant products rises daily, more synthetic dyes are used, increasing the amount of wastewater generated by the dye industries, which include textile manufacturing, leather processing, and paper printing [1, 2]. Approximately 20% of dyes in the industry end up in industrial wastewater because the color molecules are not properly fixed to the fibers, and the dyeing units are inefficient enough [3]. The discharge of those pollutants to the environment poses potential threats to the quality of water and induces serious health problems for humans, plants, and animal life in particular. Furthermore, wastewater generated by the organic dyes contains a significant amount of color, and toxic synthetic mixtures are also responsible for the clarity and taste of water, making it unpleasant to consumers [4].

Methylene blue (MB) is a heterocyclic aromatic compound mostly used to color cotton and silk, and it is quite dangerous when it is found in wastewater. Due to its high water solubility, MB causes pollution in aquatic, human, and animal food chains when discharged directly into the water without prior treatment [5]. Numerous severe health issues, including skin and eye irritations, dyspnea, burning sensations, chest pain, cancer, cyanosis, and vomiting, can result from consuming large amounts of MB [4].

In light of these challenges, methylene blue (MB) has been widely used as a model dye to evaluate the remediation performance of various materials and treatment methods, and its elimination from industrial effluents remains a high research priority to minimize the risks to the environment. Consequently, numerous techniques have been investigated for its effective removal, including ozonation, the Fenton process, sonolysis, ultrafiltration, chemical coagulation, adsorption, flocculation, oxidation, and photocatalysis [6, 7].

Among these, a clean and eco-friendly technique for eliminating numerous organic contaminants is the photocatalytic process, which transforms them into less hazardous compounds without producing any secondary pollutants. Additionally, photocatalytic degradation methods need low technical complexity, high stability, low operating cost, and ambient operating conditions, making them the most promising compared to conventional methods [8–10].

However, in the photodegradation process, selecting a photocatalytic material with suitable optical and electronic properties is crucial, particularly its ability to absorb a broad range of light and efficiently generate and separate photogenerated electron-hole pairs, which are major challenging tasks for researchers. Because of its straightforward mechanism and experimental design, visible light-driven photocatalysis is receiving greater attention and provides more efficient photodecomposition by utilizing a wider range of the light spectrum [11].

The well-researched semiconductor materials used for photocatalysis, like TiO_2_ and ZnO, have large band gaps that limit their ability to absorb photons of visible light [12], which makes up over 46% of the solar light, as compared to UV radiation containing only 4% [13].

Due to the nontoxicity, low cost, and ease of synthesis and its ability to efficiently absorb visible light, graphitic carbon nitride (g-C_3_N_4_) photocatalysts have generated significant interest in the scientific community, where researchers are making a concerted effort to determine the unique properties that make these catalysts truly exceptional [14].

However, pristine g-C₃N₄ suffers from several limitations in photocatalytic applications, including rapid recombination of photogenerated charge carriers, limited charge-carrier mobility that restricts efficient charge transfer, a relatively low specific surface area, and poor stability under prolonged photocatalytic conditions, where continuous exposure to light and reactive species gradually degrades its performance, thereby diminishing its photocatalytic efficiency [15].

To address those shortcomings, many methods have been adopted, including loading of mono- and bimetallic NPs and the formation of heterostructures with other semiconductor materials [16]. This approach improves catalyst stability and reduces charge recombination through interfacial charge carrier transfer [17].

Plasmonic metal deposition is an effective strategy that not only modifies the electronic band structure of pure g-C_3_N_4_ but also impacts the surface properties and improves the photocatalytic performances [18].

Among these plasmonic materials, Ag exhibits strong and broad surface plasmon resonant (SPR) absorption in the visible region of the solar spectrum, with the frequency of the SPR band being strongly correlated with the size, shape, and structure of these plasmonic materials [19]. Although they possess excellent photocatalytic properties, their high cost limits the practical use of Ag nanoparticles (NPs) in photocatalysis, and the combination of Ag with less costly metals such as Cu decreases the material costs and improves the optical and catalytic properties of bimetallic NPs [20].

Although the deposition of bimetallic (NPs) on the surface of g-C₃N₄ improves photocatalytic performance, the relatively low specific surface area and poor dye adsorption capacity of g-C₃N₄ and metal NPs limit their overall photocatalytic efficiency [21].

To overcome these challenges, researchers are exploring various supporting materials that can improve stability and catalytic activity and lower the amount of NPs released during photocatalytic processes [22].

Natural clay minerals have gained significant attention as NP supports because they are more cost-effective and environmentally friendly compared to many other support materials, such as graphene and carbon nanotubes [23]. Kaolin clay, in particular, is recognized for its high silicon oxide and alumina content, which makes it an excellent support material for metal NPs. This is attributed to the strong interaction between metal ion and kaolin, as well as its high surface area and good metal dispersion [24]. Furthermore, the kaolin support can adsorb pollutants and position them close to the photocatalyst surface, where hydroxyl (OH) radicals are concentrated. This will enhance the overall efficiency of the photodegradation process [25].

Despite the considerable progress achieved in developing mono- and Ag-Cu/g-C₃N₄-based photocatalysts for wastewater treatment and other applications [26–29], the fabrication of kaolin-supported Ag-Cu/g-C₃N₄ photocatalysts through a simple and cost-effective synthesis route has received limited attention. In the present study, an Ag-Cu/g-C₃N₄/kaolin photocatalyst was synthesized via a facile chemical reduction method using naturally abundant kaolin clay as a supporting material. This work lies in integrating the adsorption capability of kaolin with the photocatalytic properties of the Ag-Cu/g-C₃N₄ catalyst to achieve enhanced photocatalytic performance under natural sunlight while employing a simple, low-cost, and environmentally benign synthesis approach. The kaolin support is expected to improve the dispersion of Ag-Cu and g-C₃N₄, suppress NPs aggregation, increase the availability of active sites, and facilitate the separation and transfer of photogenerated charge carriers.

The structural, morphological, and optical characteristics of the synthesized materials were carefully examined utilizing a variety of techniques. Then, using methylene blue (MB) dye as a typical organic pollutant, their photocatalytic efficiency was assessed under the irradiation of natural sunlight. Furthermore, a thorough investigation was conducted into the effects of catalyst dosage, solution pH, and dye concentration on degradation efficiency.

## Experimental section

### Chemicals

The kaolin clay, a natural mineral used as a support for metal NPs, was collected from Gasay, South Gondar Zone, Amhara Region, Ethiopia. This area is known for its rich deposits of white clay, making it an ideal source for kaolin used in various catalytic and adsorption applications [30]. The chemicals used included urea (CH_4_N_2_O, 99%, Sigma-Aldrich), copper sulfate pentahydrate (CuSO_4_⋅5H_2_O, 99.99%, UniChem Chemicals), silver nitrate (AgNO_3_, 99.9% LOBA CHEMIE), methylene blue (MB, C_16_H_18_ClNS, Dallul Pharmaceuticals plc), sodium borohydride (NaBH_4_, 97%, Alpha Chemika), ascorbic acid (AA, C_6_H_8_O_6_, 99%, Sigma-Aldrich), ethylenediaminetetraacetic acid disodium salt (EDTA, C_10_H_14_N_2_Na_2_O_8_, 99%, Sigma-Aldrich), isopropanol (IPA, C_3_H_8_O, 99.5%, Sigma-Aldrich), and hydrochloric acid (HCl, 37%, Sigma-Aldrich). All the reagents were of analytical grade and used without further purification.

### Wet beneficiation of kaolin clay

Physical separation and wet beneficiation processes were used to remove impurities from the raw kaolin clay, in accordance with the procedure described by Ayalew [31]. First, the dried natural kaolin clay was ground into a fine powder using a mortar and pestle, and then it was sieved using a 75 μm sieve. For beneficiation processes, 70 g of finely ground kaolin clay was soaked in 1 L of deionized water, and it was stirred with a magnetic stirrer for 24 h at room temperature.

Following the 24 h beneficiation process, the suspension was left to settle for 12 h, then separated by filtration and repeatedly washed with deionized water. The resulting solid product was dried overnight in an oven set to 70 °C and finally ground and sieved for later use.

### Synthesis of g-C_3_N_4_

With slight modification to previously reported procedures [32], g-C_3_N_4_ was produced by sequential heating of urea. Briefly, first the crucible was filled with 25 g of urea, and then it was sealed with many layers of aluminum foil. After that, it was put into a muffle furnace, which was heated to 300 °C for one hour and then to 500 °C for two hours. After cooling to room temperature, aluminum foil was removed from the crucible, a pale-yellow product was obtained, ground to a powder, and then collected for later use.

### Synthesis of kaolin supported Ag-Cu/g-C_3_N_4_ NCs

The deposition of Ag and Cu NPs on the surface of g-C₃N₄ and beneficiated kaolin support was carried out through a chemical reduction process [33]. First, we dissolved 0.37 g of g-C₃N₄ and 0.6 g of beneficiated kaolin in 25 mL of deionised water. After it was stirred for 15 min, 25 mL of 0.0556 M AgNO₃ and 25 mL of 0.0944 M CuSO₄⋅5 H₂O solutions were added, then it was stirred for 2 h at room temperature. To convert Ag⁺ and Cu²⁺ to their corresponding NPs, 25 mL of a 0.2 M NaBH₄ solution was added slowly while being stirred rapidly. After it was stirred for an additional 40 min, the obtained precipitate was centrifuged and washed several times with deionised water to eliminate any leftover NaBH₄ and other ions. Finally, the precipitate was dried in an oven set at 60 °C overnight.

**Scheme 1:**
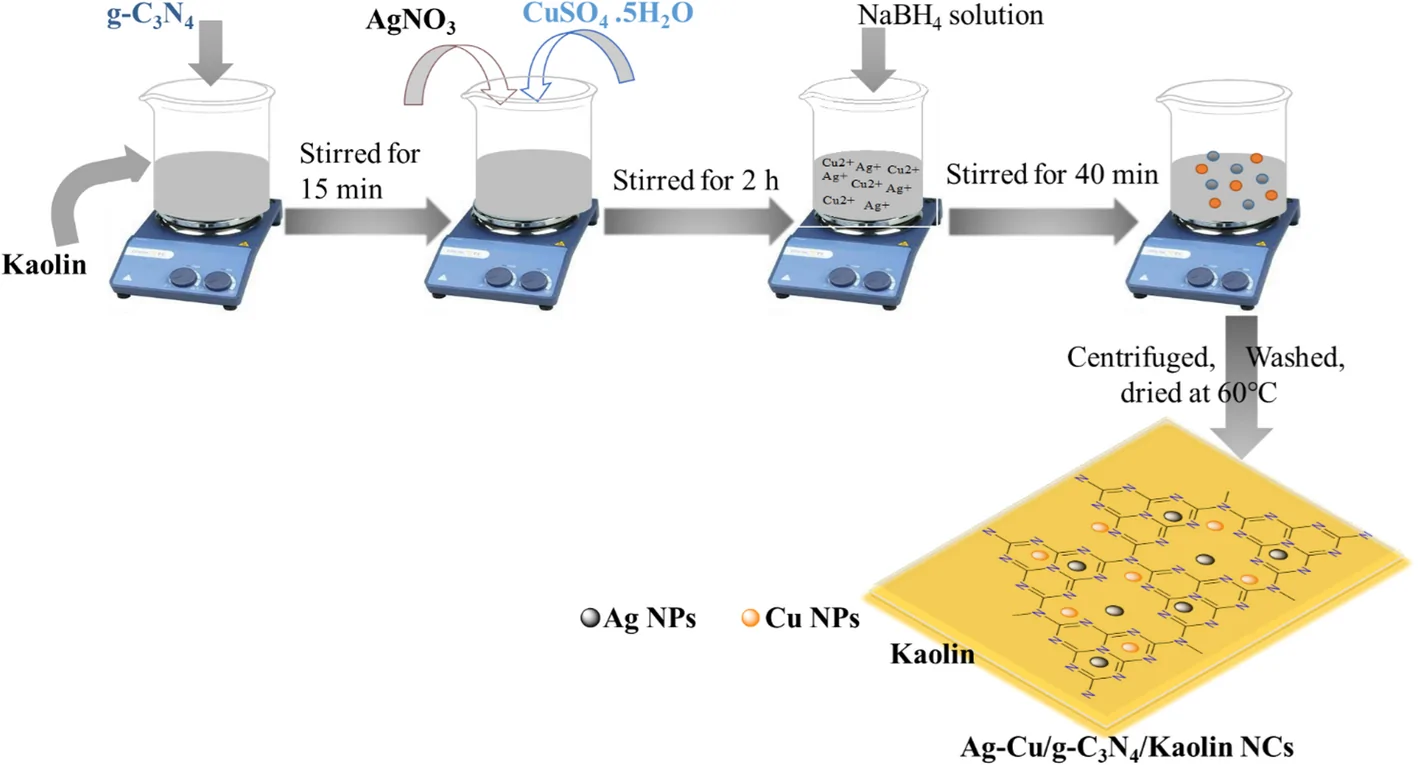
Synthesis pathway for Ag-Cu/g-C_3_N_4_/kaolin NCs

To prepare Ag–Cu/g-C₃N₄/kaolin NCs with different Ag–Cu/g-C₃N₄ ratios, the same procedures were repeated three times, following the same path using different quantities of AgNO₃, CuSO₄·5 H₂O, and g-C₃N₄, while keeping the kaolin quantity constant at 0.6 g. Then, the prepared samples were labelled as 1-Ag–Cu/g-C₃N₄/kaolin, 3-Ag–Cu/g-C₃N₄/kaolin, and 5-Ag–Cu/g-C₃N₄/kaolin for 1%, 3%, and 5% Ag–Cu, respectively.(Scheme 1)

### Characterization

The synthesized samples were characterized to study their optical, structural, morphological, compositional, and surface properties using several instruments. The ultraviolet−visible (UV–vis), Fourier transform infrared (FT-IR), X-ray diffraction (XRD), scanning electron microscopy (SEM), energy dispersive x-ray spectroscopy (EDS), transmission electron microscopy (TEM), high-resolution transmission electron microscopy (HRTEM), and X-ray photoelectron spectroscopy (XPS) were used.

### Photocatalytic experiment

To test the photocatalytic activity of the Ag-Cu/g-C₃N₄/kaolin NC, we added a certain amount of the Ag-Cu/g-C₃N₄/kaolin sample to a 50 ml (10 mg/L) aqueous solution of MB dye using a 100 mL glass beaker as the reaction vessel. Before the photocatalytic degradation experiment, the solution was stirred for 30 min in the dark to establish adsorption-desorption equilibrium. Afterward, the reaction suspension was exposed to natural sunlight from approximately 11:50 a.m. to 4:30 p.m. under continuous stirring. This time was selected to utilize the relatively high solar irradiance typically available around midday and the early afternoon, while maintaining consistent experimental conditions as much as possible. After each 20 min of stirring time, 3 mL of an aliquot of the sample was taken out, and it was centrifuged to separate the photocatalyst. Then a UV-visible double-beam spectrophotometer was used to evaluate for changes in intensity associated with the λmax of MB, which appears at 664 nm.

Equations 1 and 2 given below were used to calculate the MB degradation efficiency and reaction rate constant, respectively [34].

1 $$\:\mathrm{D}\mathrm{e}\mathrm{g}\mathrm{r}\mathrm{a}\mathrm{d}\mathrm{a}\mathrm{t}\mathrm{i}\mathrm{o}\mathrm{n}\:\mathrm{e}\mathrm{f}\mathrm{f}\mathrm{i}\mathrm{c}\mathrm{i}\mathrm{e}\mathrm{n}\mathrm{c}\mathrm{y}\:\left(\%\right)=\:\left(\frac{{\mathrm{A}}_{0}-{\mathrm{A}}_{\mathrm{t}}}{{\mathrm{A}}_{0}}\right)\:\mathrm{x}\:100\mathrm{\%}$$

2 $$\:\mathrm{ln}\left(\frac{{\mathrm{A}}_{\mathrm{t}}}{{\mathrm{A}}_{0}}\right)=-\mathrm{k}\mathrm{t}$$

where $\:{\mathrm{A}}_{0}$ is the absorbance at the initial time, and $\:{\mathrm{A}}_{\mathrm{t}}$ is the absorbance after each irradiation time.

### Scavenging experiment

To identify the reactive species responsible for MB degradation, different trapping tests were performed using ascorbic acid, isopropanol alcohol, and EDTA-2Na to quench superoxide radicals, hydroxyl radicals, and holes, respectively. To perform this experiment, a mixture consisting of 50 mL of 10 mg/L MB, 15 mg of Ag-Cu/g-C_3_N_4_/kaolin NCs, and 5 mM scavenging solution was exposed to sunlight for 120 min while being stirred continuously, and photodegradation was monitored by taking UV-Vis absorption data with a time interval of 20 min.

### Recovery and reuse

One of the main key factors that make the wastewater treatment process attractive is the ability to be regenerated, recovered, and reused after serving as catalysts. Nanomaterials with high recovery and regeneration capabilities are highly valued, as they can significantly reduce overall treatment costs [35].

To evaluate the reusability of 3-Ag-Cu/g-C_3_N_4_/kaolin NC, the successive photocatalytic degradation experiments under sunlight irradiation were conducted. For the first cycle, 15 mg of Ag-Cu/g-C_3_N_4_/kaolin NC was added into 50 mL of 10 mg/L MB solution, and it was exposed to sunlight irradiation for 120 min with continuous stirring. After each cycle, the NC was recovered through centrifugation, washed several times with deionized water, and dried at 80℃. The recovered NC was then reused in subsequent cycles under the same experimental conditions.

## Results and discussion

### FT-IR and XRD analysis

**Fig. 1:**
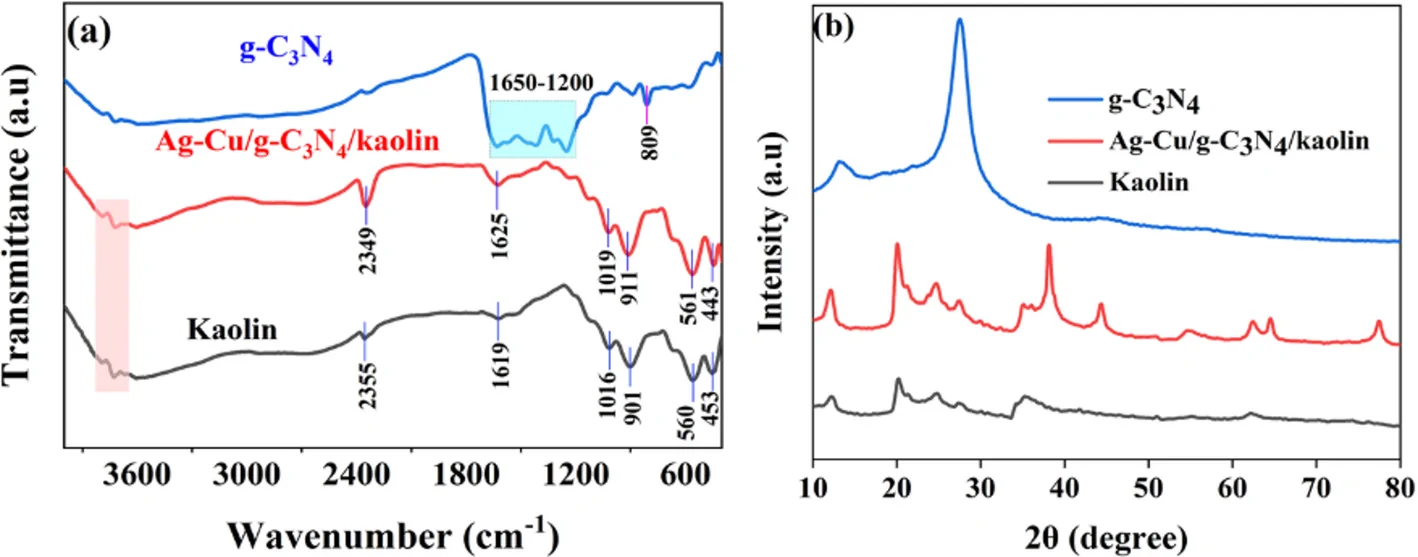
FTIR (**a**) and XRD (**b**) spectra of g-C_3_N_4_, kaolin, and 3-Ag-Cu/g-C_3_N_4_/kaolin NC

FT-IR spectroscopy is used to analyze the chemical compositions and functional groups of the samples. The FT-IR spectra of the g-C_3_N_4_, kaolin, and 3-Ag-Cu/g-C_3_N_4_/kaolin NC recorded in the 400–4000 cm^− 1^ range are shown in Fig. 1a.

In the FTIR spectrum of g-C_3_N_4_, the peaks observed between 1200 and 1650 cm^− 1^ are responsible for the stretching vibration modes of the C = N and C–N functional groups in the heterocyclic structure [36], whereas the characteristic peak at around 809 cm^− 1^ corresponds to out-of-plane bending vibration of the s-triazine ring, indicating the proper formation of the g-C_3_N_4_ phase [37]. These results are in good agreement with the previously reported works [38, 39].

The FTIR spectrum of beneficiated kaolin illustrates the absorption band between 3600 and 3750 cm^− 1^ corresponding to OH stretching vibrations, while absorption bands between 1620 and 1720 cm^− 1^, are due to the H–O–H mode of water molecules [31], while the absorption bands at 1019 cm^− 1^, 1150 cm^− 1^ and 911 cm^− 1^ are due to the Si–O–Si group of the tetrahedral sheet, Si–O stretching vibration, and Al–OH, respectively. This demonstrates that a significant amount of silicon oxide was present in the kaolin under study [40]. Furthermore, the presence of aluminum oxide next to silicon oxide is also indicated by a distinct and sharp peak at 562 cm^− 1^ that correlates with Si-O-Al bending vibration [41].

Almost all these beneficiated kaolin peaks are also observed in 3-Ag-Cu/g-C_3_N_4_/kaolin NC, suggesting that the incorporation of Ag-Cu NPs and g-C_3_N_4_ does not alter the chemical structure of kaolin clay, which indicates that the chemical framework of beneficiated kaolin remains stable after the introduction of Ag-Cu NPs and g-C_3_N_4_. However, in 3-Ag-Cu/g-C_3_N_4_/kaolin NC, a small shift in peak position is observed, indicating the interaction of Ag-Cu and g-C_3_N_4_ with kaolin [42].

The crystal structure of the prepared samples was analyzed by XRD. According to the XRD patterns of beneficiated kaolin (Fig. 1b), several peaks characterizing kaolin were detected. The diffraction peaks at 2θ values of 12.24°, 20.38°, 23.75°, 35.33°, and 62.15° were indexed to the Miller indices of (001), (100), (002), (110), and (300), respectively [43, 44]. Additionally, a peak assigned to quartz appeared at about 27.53°, confirming that the quartz material was not completely removed through beneficiation, resulting in increasing the silicon dioxide (SiO_2_) content (31).

The XRD of g-C₃N₄ shows two typical diffraction peaks at 12.8° and 27.7°, which correspond to (100) and (002) planes, indicating the in-plane structural arrangement of the heptazine units of g-C₃N₄ and the layered stacking structure of the sheets of g-C₃N₄, respectively [45].

In the Ag-Cu/g-C₃N₄/kaolin NC, the diffraction peaks observed at 2θ = 38.5°, 44.5°, 64.7°, and 77.7° can be indexed to the (111), (200), (220), and (311) crystal planes, respectively, and may be attributed to metallic Ag (JCPDS 04-0783) [26], confirming the presence of crystalline Ag in the NC. XRD peaks corresponding to Cu were detected, which perhaps was the result of the amorphous nature [46] or due to the small size and uniform dispersion [47] of Cu NPs over the kaolin surface. However, the existence of Cu NPs was confirmed from EDX and XPS analysis. Ag-Cu/g-C₃N₄/kaolin NC retains several characteristic reflections of kaolin, indicating that the incorporation of g-C₃N₄ and Ag/Cu did not completely destroy the crystalline mineral framework. Interestingly, the XRD patterns of Ag-Cu/g-C₃N₄/kaolin NC do not show the diffraction peaks corresponding to g-C₃N₄. This could be because the high dispersion of g-C₃N₄ nanosheets on the surface of kaolin causes the layer stacking pattern to disappear [48].

The crystallite size of the Ag phase in 3-Ag/Cu/g-C₃N₄/kaolin NC was estimated using the Scherrer Eqs. [49, 50]. and was approximately 11.99 nm.

### UV–vis analysis

**Fig. 2:**
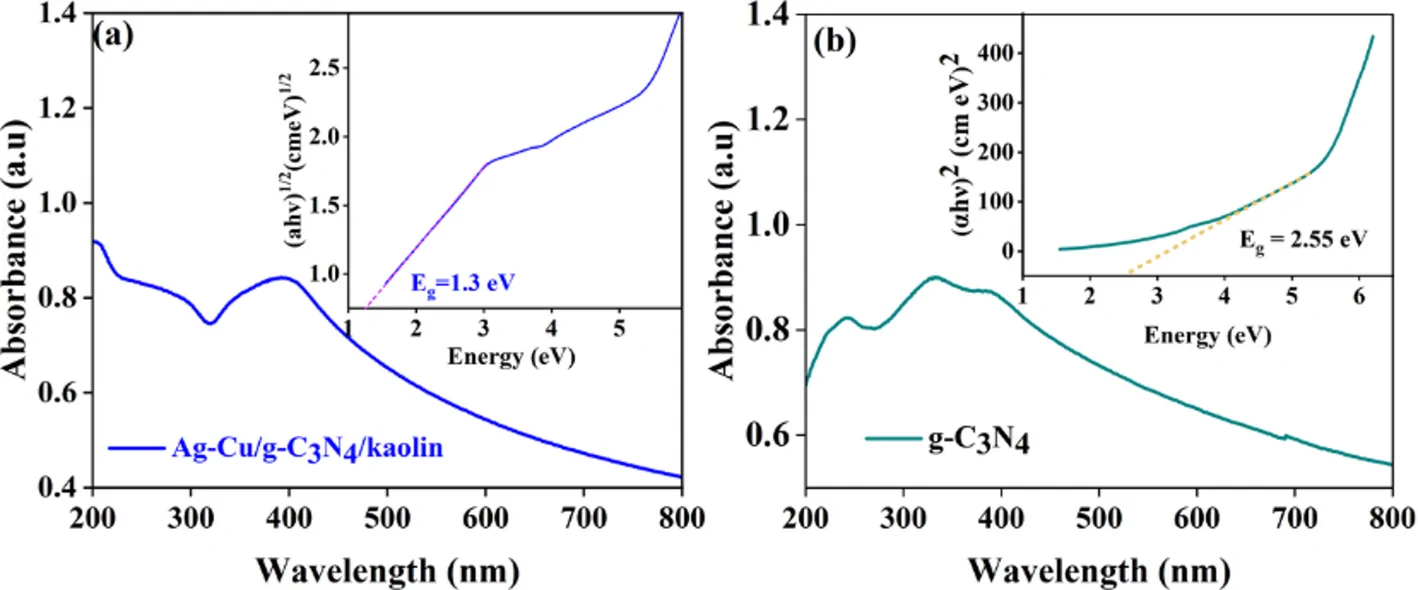
UV-vis absorption spectra of 3-Ag-Cu/g-C_3_N_4_/kaolin NC and g-C_3_N_4_ samples with insets of Tauc plots and the estimated bandgap energies

The light absorption characteristics of a catalyst have a significant influence on the photocatalytic behavior, and the extent of its photo response is a crucial factor for its photocatalytic efficiency [51]. The pure g-C_3_N_4_ (Fig. 2b) exhibits an absorption peak centered at 330 nm with an absorption edge around 480 nm, indicating that g-C_3_N_4_ is inefficient at utilizing the entire visible light spectrum [52]. In comparison, the 3-Ag-Cu/g-C₃N₄/kaolin NC exhibits a broader absorption profile and a shift of the absorption edge toward longer wavelengths relative to pure g-C₃N₄ (Fig. 2a). Thus, the light absorption spectrum is broadened, which is beneficial for the generation of more electron-hole pairs, in turn improving the photocatalytic activity [26].

Band gap energies of the samples were estimated from Tauc plots (Eq. 3). This involves plotting (𝛼ℎ𝜈)^n^ against ℎ𝜈 and then extrapolating the linear portion of the curve to the energy axis. The intercept obtained from this extrapolation corresponds to the estimated band gap energy of the material [36], and it was determined that the band gaps of the 3-Ag-Cu/g-C_3_N_4_/kaolin NC and g-C_3_N_4_ were 1.3 eV and 2.55 eV (inset of Fig. 2a, b), respectively.

3 $$\:{\left({\upalpha\:}\mathrm{h}{\upupsilon\:}\right)}^{\mathrm{n}}=\mathrm{A}\left(\mathrm{h}{\upupsilon\:}-{\mathrm{E}}_{\mathrm{g}}\right)$$

where α, h, ν, A, and E_g_ represent the optical absorption coefficient, Planck constant, photon frequency, a constant, and band gap energy, respectively, and n denotes the type of electronic transitions.

### SEM and EDS

**Fig. 3:**
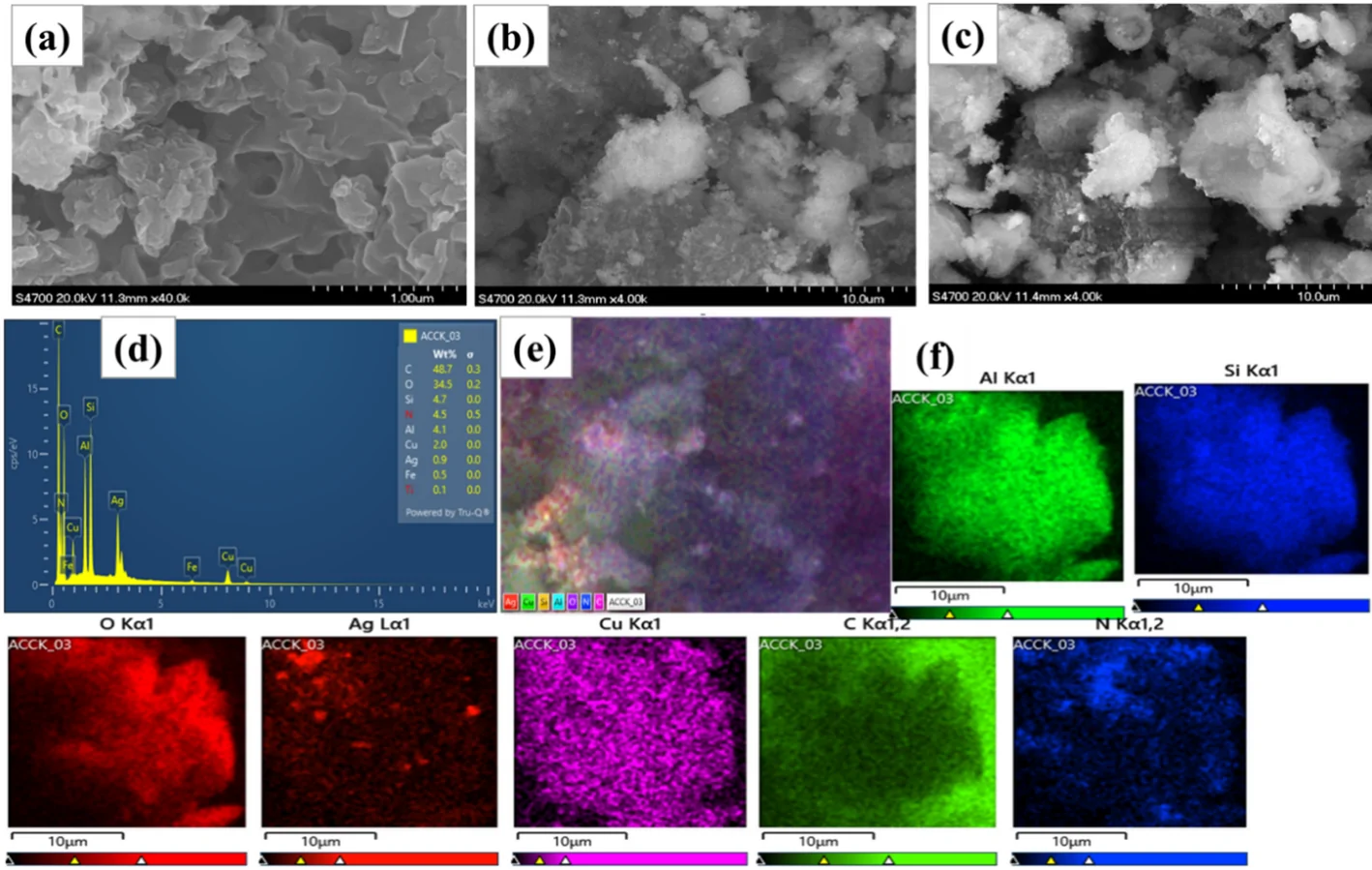
SEM micrograph of g-C₃N₄ (**a**), beneficiated kaolin (**b**), 3-Ag-Cu/g-C₃N₄/kaolin NC (**c**), and EDS spectrum of 3-Ag-Cu/g-C₃N₄/kaolin NC (**d**); the corresponding merged elemental map (**e**) and individual elemental maps (**f**)

The surface morphology of the g-C_3_N_4_, beneficiated kaolin, and Ag-Cu/g-C_3_N_4_/kaolin NC were investigated using scanning electron microscopy (SEM), as illustrated in Fig. 3. The SEM morphology of g-C_3_N_4_ (Fig. 3a) exhibits irregular solid agglomerates of stacked flakes with some large voids across the surface. This morphology indicates the successful exfoliation of g-C_3_N_4_ layers from the bulk material [53]. A flaky, sheet-like morphology was observed in the SEM images of kaolin, with certain illuminated plates suggesting the presence of quartz [54]. Additionally, because large particles are ground into smaller ones and then aggregate together, small aggregates can be seen on the kaolin surface [55]. After being impregnated with Ag-Cu NPs and g-C_3_N_4,_ the kaolin layer became very rough, fragmented, and highly porous (Fig. 3c). This morphological change indicates the incorporation of Ag-Cu NPs and g-C_3_N_4_, which modifies the surface texture of kaolin support. The increased porosity may provide more accessible surface sites for dye adsorption and photocatalytic reactions, potentially contributing to the enhanced photocatalytic degradation efficiency. Furthermore, the presence of the constituent elements C, O, Si, N, Al, Cu, and Ag with a weight% of 48.7, 34.5, 4.7, 4.5, 4.1, 2.0, and 0.9%, respectively, is also verified by EDS (Fig. 3d); this provides strong evidence that 3-Ag-Cu/g-C₃N₄/kaolin NC was successfully synthesized. The trace amounts of Fe (0.5%) and Ti (0.1%) may be attributed to minor impurities found in the naturally occurring kaolin. The higher carbon content observed in the SEM/EDS may result from carbon tape used for sample mounting and adventitious carbon contamination [56].

### TEM and HRTEM

**Fig. 4:**
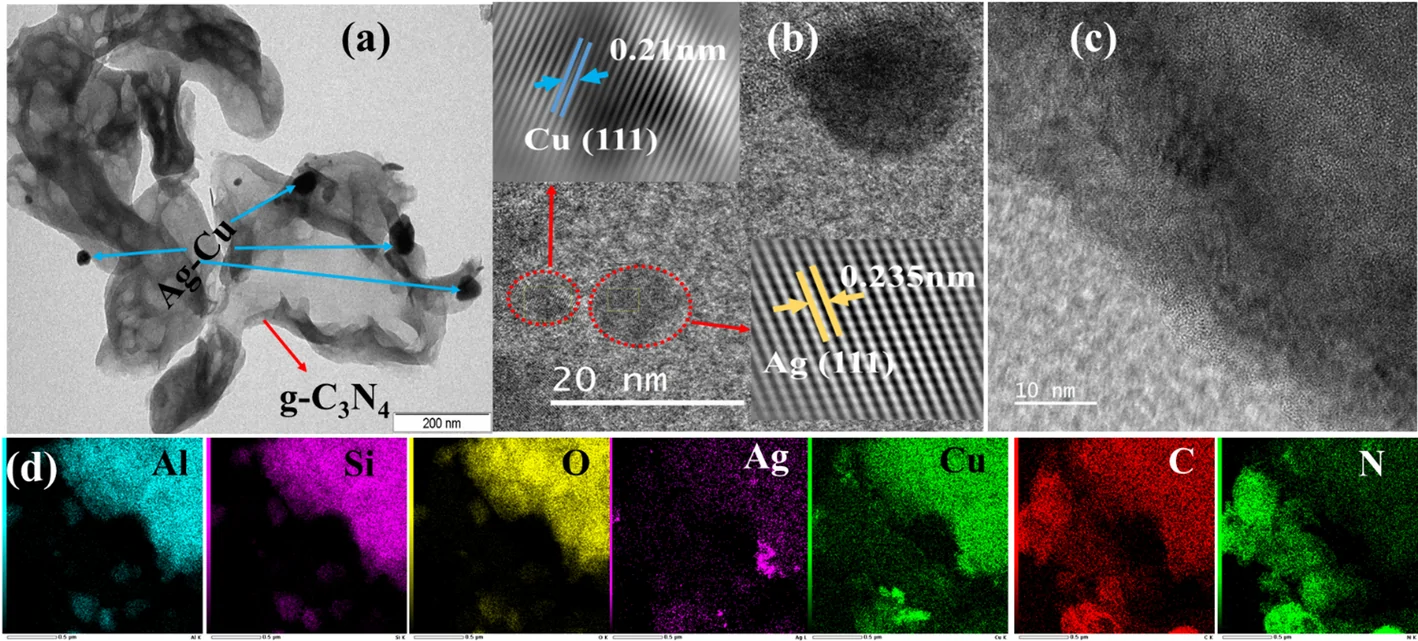
TEM image (**a**), HRTEM image with a 20 nm scale bar and an enlarged inset showing the lattice fringes and corresponding interplanar spacing (**b**), HRTEM image with a 10 nm scale bar (**c**), and elemental mapping analysis (**d**) of the 3-Ag-Cu/g-C₃N₄/kaolin NC

The morphological characteristics of the prepared sample were investigated using TEM analysis. As shown in Fig. 4a, the TEM images of the 3-Ag-Cu/g-C_3_N_4_/kaolin NC show distinct small black NPs dispersed on the surface of kaolin. This result indicates the successful dispersion of Ag-Cu NPs over the kaolin surface.

To further elucidate the morphology and lattice spacing of the prepared 3-Ag-Cu/g-C₃N₄/kaolin NC, HRTEM with 20 nm resolution was employed. As shown in Fig. 4b, two distinct sets of lattice fringes with inter-planar spacing of approximately 0.235 and 0.2 nm were observed, which can be assigned to the Ag (111) and Cu (111) planes, respectively. These lattice-fringe measurements provide further evidence for the presence of Ag and Cu in the prepared NC. However, the lattice fringes of g-C₃N₄ were not identified in the NC due to the ultrathin layer structure or poor crystallinity [18, 57]. To further examine the elemental information of the NC, EDX analysis was carried out, and the result showed the presence of Al, Si, O, Ag, Cu, C, and N. Therefore, these results evidently endorse that the Ag-Cu NPs are combined with the 2D sheet-like structures of g-C_3_N_4_ over the surface of kaolin, which are anticipated to enhance catalytic activity.

### XPS

**Fig. 5:**
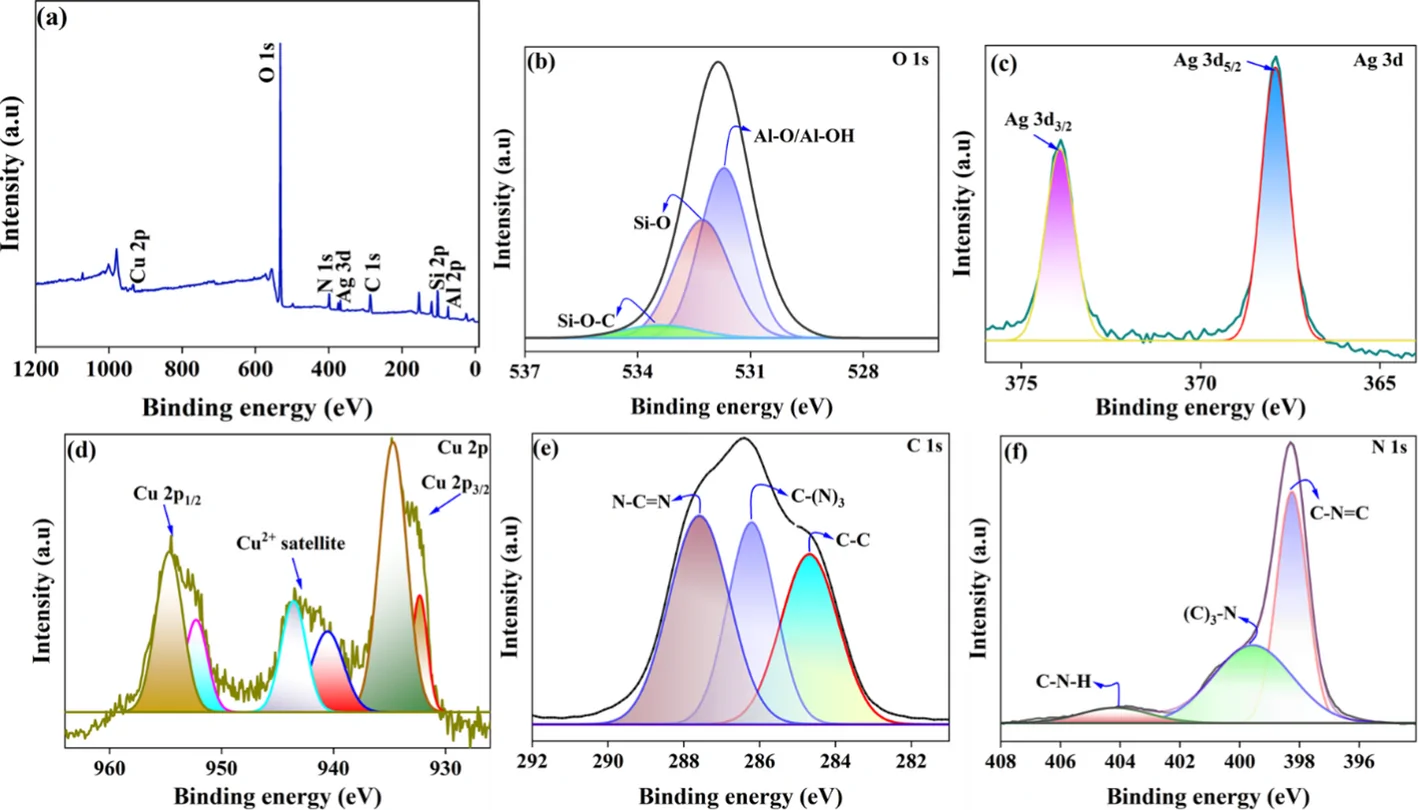
XPS survey spectra of 3-Ag-Cu/g-C_3_N_4_/kaolin NC (**a**), XPS core level spectra of O 1s (**b**), Ag 2p (**c**), Cu 2p (**c**), C1s (**d**), and N 1s (**e**)

XPS analysis was used to analyze the chemical states, elemental composition, and electronic properties of the 3-Ag-Cu/g-C_3_N_4_/kaolin NC. The survey spectra (Fig. 5a) confirm the presence of all the composite constituents: silver (Ag 3d), copper (Cu 2p), carbon (C 1s), nitrogen (N 1s), silicon (Si 2p), aluminum (Al 2p), and oxygen (O 1s).

The O1s XPS spectra were deconvoluted into three components (Fig. 5b); the first component placed at binding energies of 531.7 eV was attributed to lattice oxygen [58], such as Al-O and Al-OH. The second peak placed at 532.3 eV related to Si-O, and the third peak centered at 533.2 eV was due to Si–O–C groups. The formation of the Si–O–C group proved that kaolin and g-C_3_N_4_ were successfully compounded together during the synthesis processes [59]. As shown in Fig. 5c, the Ag 3d scan reveals the presence of Ag NPs through the deconvolution of Ag 3d_5/2_ and Ag 3d_3/2_ spectra at binding energies of 367.9 and 373.9 eV, respectively, with a consistent spin–orbit splitting of 6.00 eV [60]. Additionally, the shape and binding energy positions are characteristic of metallic Ag⁰, as similar results have been reported in the XPS literature [61].

The high-resolution Cu 2p XPS spectrum is deconvoluted into six components (Fig. 5d) with binding energies at about 932.3, 934.6, 940.5, 943.3, 952.2, and 954.4 eV. The peaks at 932.3 and 934.6 eV could be assigned to the Cu 2p₃/₂ components of Cu⁰/Cu⁺ and Cu²⁺ species, respectively [62], while the peaks at 952.2 and 954.4 eV were attributed to the Cu 2p₁/₂ components of Cu⁰/Cu⁺ and Cu²⁺, respectively. The well-known shake-up satellite features observed at 940.5 and 943.3 eV are typical for Cu²⁺ species and provide further evidence for the presence of Cu²⁺ on the catalyst surface [63]. The energy separation between the Cu 2p₃/₂ and Cu 2p₁/₂ components is approximately 20 eV, which is consistent with the characteristic spin–orbit splitting of Cu 2p [64].

As shown in Fig. 5e, for C1s, three peaks were detected at 284.7, 286.2, and 287.6 eV. The first peak was associated with the C–C bonds, the second with C–(N)_3_ bonds, and the last with a typical signal for sp²-hybridized carbon, which was identified as the C–N = C backbone of g-C₃N₄ [59]. The high-resolution N1s spectra could be divided into three peaks (Fig. 5f). The main peaks centered at 398.8 eV and 399.6 eV may be caused by the sp^2^-hybridized nitrogen (C–N = C) in the triazine rings and the bridging nitrogen atoms (C)_3_–N, respectively [65]. The additional weak peak observed at approximately 404.2 eV may be caused by the amino functional groups with hydrogen (C–N–H) and may be connected to incomplete condensation, which is commonly observed in the N 1s XPS spectrum [66].

### Photocatalytic performance of Ag-Cu/g-C_3_N_4_/Kaolin NCs

The photocatalytic activity of g-C_3_N_4_, g-C_3_N_4_/kaolin and Ag-Cu/g-C_3_N_4_/kaolin NC was evaluated using 50 mL of MB (10 mg/L) under exposure to natural sunlight, and the change in absorbance of MB was monitored using UV-visible spectroscopy. Prior to irradiation, dark adsorption experiments were conducted for 30 min to estimate the adsorption of MB onto the prepared photocatalysts. The adsorption removal efficiency of pure g-C₃N₄ was 13%, whereas those of g-C₃N₄/kaolin and 3-Ag-Cu/g-C₃N₄/kaolin NC were 34% and 40%, respectively.

**Fig. 6:**
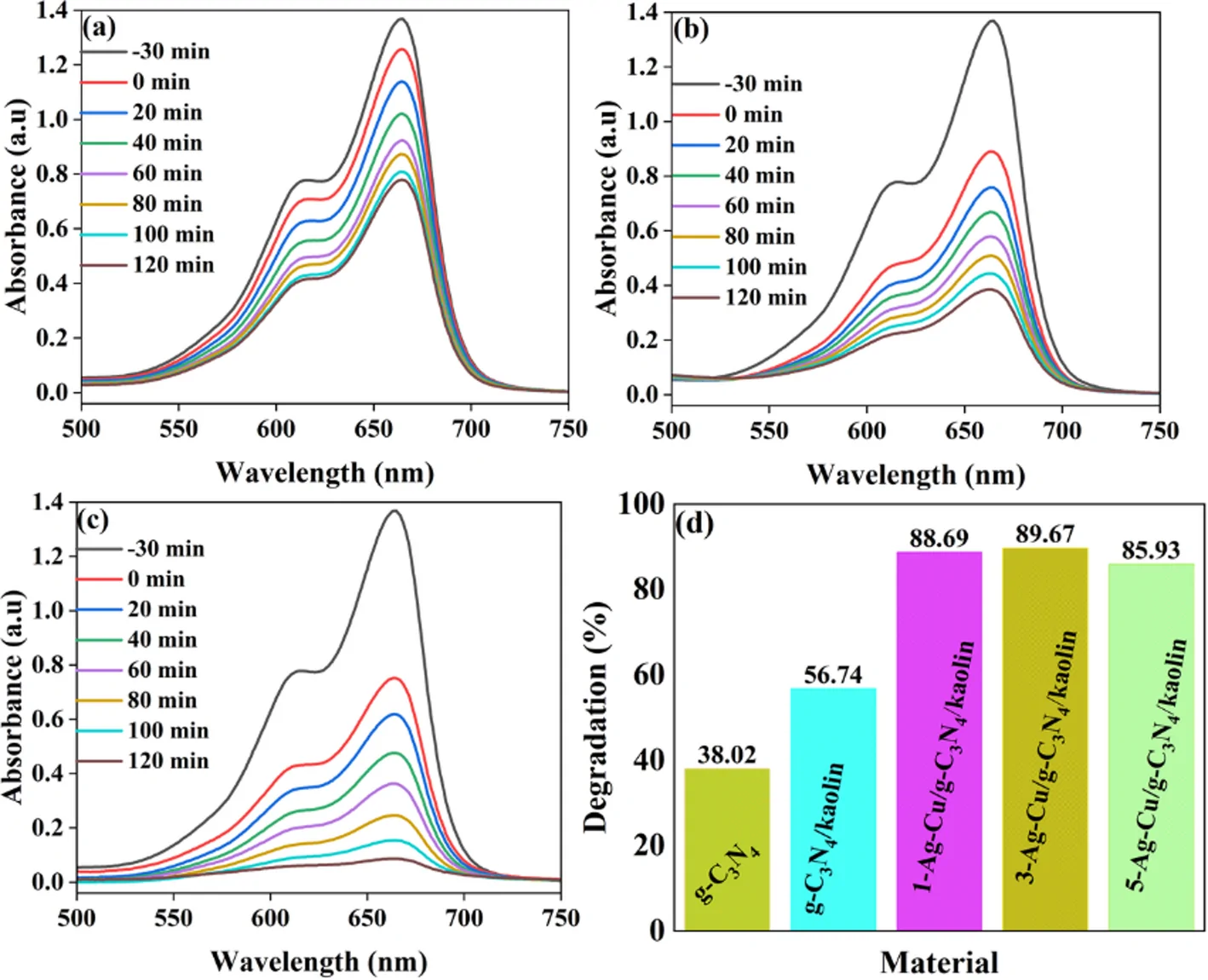
UV-Vis absorption spectra for the photodegradation of MB in the presence of g-C_3_N_4_ (**a**), g-C_3_N_4_/kaolin NC (**b**), 3-Ag-Cu/g-C_3_N_4_/kaolin NC (**c**), and the degradation efficiency of different prepared catalysts (**d**)

Under sunlight irradiation, 3-Ag-Cu/g-C_3_N_4_/kaolin NC exhibited superior photocatalytic performance, achieving 89.67% degradation efficiency of MB within 120 min of irradiation, compared to 56.74% for g-C_3_N_4_/kaolin and 38.02% for pristine g-C_3_N_4_ (Fig. 6d). The surface plasmon resonance effect of Ag-Cu NPs and the charge transfer between Ag-Cu and g-C_3_N_4_ of the photocatalyst significantly encourage the production of photoinduced charge carriers while suppressing their recombination [67]. These factors are responsible for the enhanced photocatalytic performance of Ag-Cu/g-C_3_N_4_/kaolin NC.

To get more information about the catalytic performance of the prepared NCs, the apparent rate constant (k) of pseudo-first order for the photocatalytic degradation of MB dye was calculated (Table 1). The rate constant values of 3-Ag-Cu/g-C_3_N_4_/kaolin NC are 4.8 and 2.8 times higher than those of pristine g-C_3_N_4_ and g-C_3_N_4_/kaolin NC, respectively. Furthermore, to evaluate the effect of NP loading on the NCs photocatalytic activity, various ratios of Ag-Cu to g-C_3_N_4_ were used, and 3-Ag-Cu/g-C_3_N_4_/kaolin NC showed a better photocatalytic performance as compared to 1-Ag-Cu/g-C_3_N_4_/kaolin NC and 5-Ag-Cu/g-C_3_N_4_/kaolin NC.

**Table 1:** Apparent rate constants and correlation coefficients (R²) for the photodegradation of MB using different prepared catalysts

Synthesized material	Rate constant (min^− 1^)	*R* ^2^
g-C_3_N_4_	0.0040	0.977
g-C_3_N_4_/kaolin	0.0069	0.999
1-Ag-Cu/ g-C_3_N_4/_kaolin	0.0177	0.965
3-Ag-Cu/ g-C_3_N_4/_kaolin	0.0191	0.973
5-Ag-Cu/ g-C_3_N_4/_kaolin	0.0161	0.950

The higher correlation coefficient (R^2^) values for all prepared materials confirm that the photocatalytic degradation processes follow pseudo-first order kinetics, confirming a good fit to the kinetic model.

After examining the enhanced performance of the 3-Ag-Cu/g-C_3_N_4_/kaolin NC, we looked at how various factors affect the photodegradation processes, and to that end, we have investigated the effect of phototcatalyst load, initial dye concentration, and pH of the solution.

#### Effect of catalyst dosage

In order to examine the effect of photocatalyst dose in photocatalytic degradation of MB under direct natural sunlight irradiation, the amount of 3-Ag-Cu/g-C_3_N_4_/kaolin NC was changed from 5 mg to 20 mg per 50 mL of 10 mg/L MB dye solution.

**Fig. 7:**
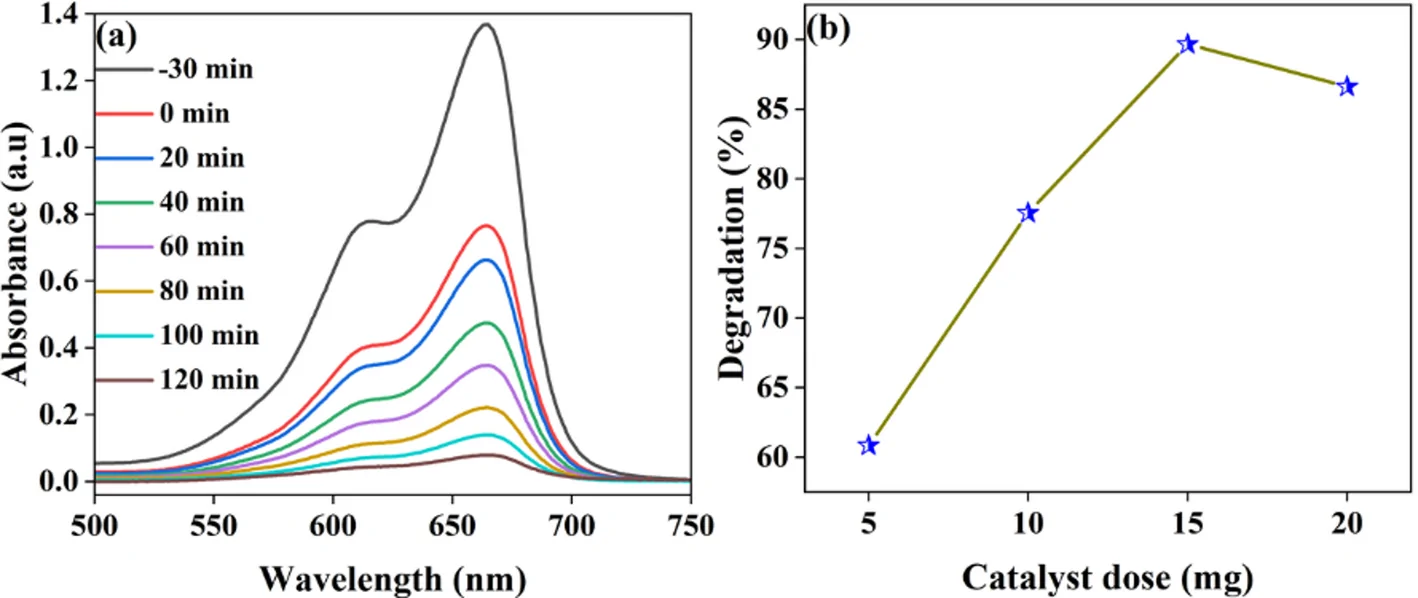
Changes in UV–Vis absorption spectra of 10 mg/L MB during photocatalytic degradation using 15 mg of 3-Ag-Cu/g-C₃N₄/kaolin NC (**a**), and MB degradation efficiency at different NC dosages after 120 min of irradiation (**b**)

Figure 7b shows the effect of catalyst quantity on the photocatalytic removal of MB dye after 120 min of reaction time. In this investigation, the degradation efficiency was increased from 60.83% to 89.67% during a 120-minute irradiation period when the photocatalyst quantity was raised from 5 mg to 15 mg. The increase in MB degradation may be due to the higher active surface area resulting from a higher catalyst dosage.

However, a slight decrease in photocatalytic degradation was observed upon raising the amount of catalyst to 20 mg. These phenomena may be explained by the fact that when a sufficiently large amount of catalyst is used, the suspension becomes more turbid, which causes light screening, decreases the formation of reactive oxygen species (ROS), and ultimately slows down the rate of dye photocatalytic degradation [68]. Another explanation for the reduction of degradation efficiency at high catalyst doses may be the formation of catalyst aggregations, which reduce the percentage of photocatalytic degradation by lowering the active site of the photocatalytic surface [69].

Similarly, the catalyst dose of 15 mg exhibited the highest apparent reaction rate constant, reaching 0.0191 min⁻¹, which is approximately 2.387 times higher than that of the 5 mg catalyst dose and 1.552 times higher than the 10 mg catalyst dose. However, as the catalyst amount increased to 20 mg, the value of the rate constant decreased to 0.0169 min^− 1^.(Table 2)

**Table 2:** Apparent rate constants and correlation coefficients (R²) for the photodegradation of MB using different doses of 3-Ag-Cu/g-C_3_N_4_/kaolin NC

Catalyst dose (mg)	Rate constant (min^− 1^)	*R* ^2^
5	0.0080	0.9836
10	0.0123	0.9959
15	0.0191	0.9732
20	0.0169	0.9863

#### Effect of initial MB concentration

To investigate the effect of the initial dye concentration on the photocatalytic degradation of MB, the initial concentrations of MB were varied from 5 mg/L to 20 mg/L, while maintaining a constant catalyst dosage of 15 mg.

**Fig. 8:**
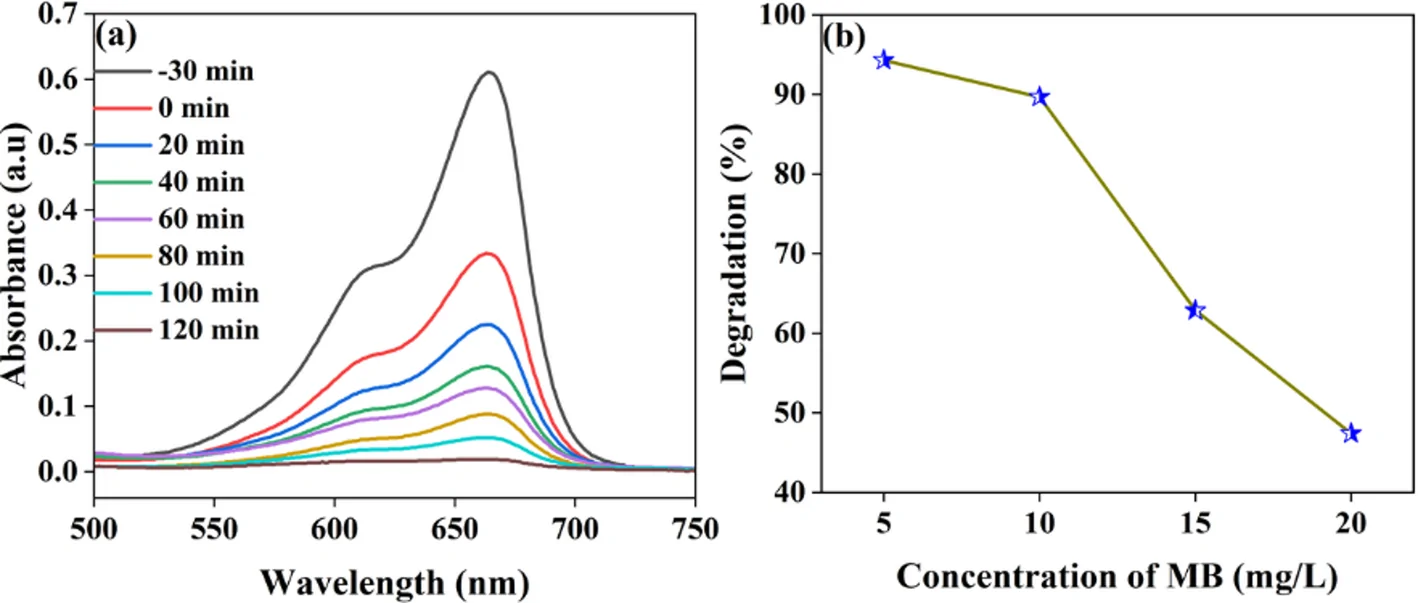
Changes in the UV–Vis absorption spectra of 5 mg/L MB during photocatalytic degradation using 15 mg of 3-Ag-Cu/g-C₃N₄/kaolin NC (**a**), and MB degradation efficiency at different initial MB concentrations after 120 min of irradiation (**b**)

Following 120 min of sunlight irradiation in the presence of 15 mg catalyst, degradation efficiencies of 94.29% and 89.67% were obtained for 5 mg/L and 10 mg/L, respectively (Fig. 8b). However, as the initial MB concentrations of the dye grew to 15 and 20 mg/L, respectively, the degradation efficiency dropped significantly, reaching 62.87% and 47.41%. The decrease in the efficiency of photodegradation with an increase in the initial dye concentration may be mainly due to the limited active sites on the surface of the catalyst. The increase in the number of dye molecules at higher MB concentrations can also cause more absorption of incident light by the dye molecules, thus reducing the amount of light reaching the catalyst surface and limiting the generation of photogenerated charge carriers [70]. Moreover, higher initial MB dye concentration causes the accumulation of reaction intermediates on the catalyst surface, which may compete with MB molecules for active sites and can slow down the degradation process.

#### Effect of pH

Determining the pH range at which the high dye degradation efficiency occurs is crucial because the pH influences the charge distribution on the surface of the photocatalyst as well as the electrostatic interactions between the MB dye and the photocatalyst surface in the aqueous solution [71]. When the photocatalyst forms a negative surface, electrostatic attraction with cationic MB dye increases, implying that the photodegradation efficiency of MB will improve. In contrast, in an acidic medium, the positively charged surface of the photocatalyst has a tendency to oppose the adsorption of cationic MB dye, which drops the rate of photocatalytic degradation [72].

To illustrate this, the photocatalytic degradation of MB in the presence of 3-Ag-Cu/g-C_3_N_4_/kaolin was investigated at pH values of 5, 7, 9, and 11. The pH of the dye solution was adjusted using either 0.1 M HCl or 0.1 M NaOH solutions. The highest percentage of dye degradation, almost 98.95%, was recorded at pH 9 with a reaction time of 120 min, which is 1.88 times more than the degradation efficiency under the pH of 5 and 1.35 times at pH 7. This enhanced degradation efficiency could be explained by the fact that higher pH causes more hydroxyl ions to be generated on the surface of the photocatalyst, which may then combine with holes to form hydroxyl radicals that could speed up the process of dye degradation [73].

**Fig. 9:**
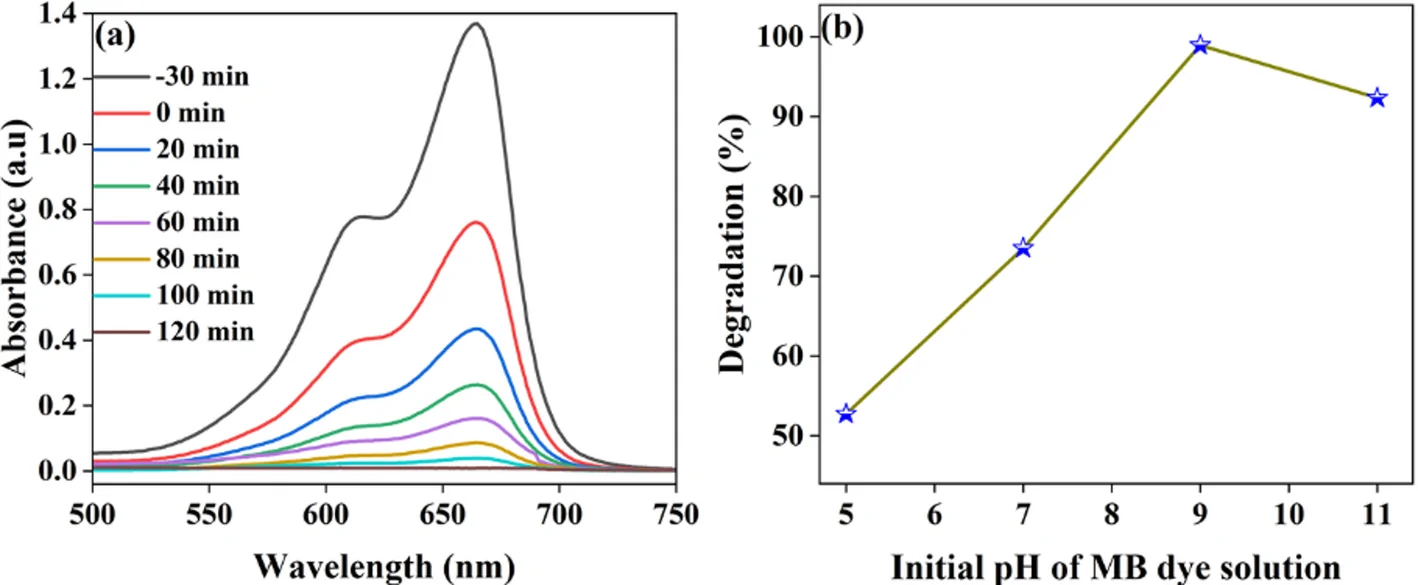
Changes in UV–Vis absorption spectra of 10 mg/L MB during photocatalytic degradation using 15 mg of 3-Ag-Cu/g-C₃N₄/kaolin NC at pH 9 (**a**), and MB degradation efficiencies at different initial pH values after 120 min of sunlight irradiation (**b**)

On the other hand, the photodegradation efficiency decreased as the pH increased to 11 (Fig. 9b). The competitive adsorption of excess hydroxyl ions on the active sites and a screening effect brought on by the high ionic concentration, which prevents MB molecules from effectively approaching the catalyst surface, are responsible for the observed reduction in efficiency [74].

#### Comparison studies

This study thoroughly assessed the MB degrading efficiency of the Ag-Cu/g-C₃N₄/kaolin NC and compared its performance with previously reported catalysts in the literature, as summarized in Table 3. This NC showed superior or comparable performance to previously reported photocatalysts, attributable to the synergistic interaction between Ag-Cu and g-C₃N₄ that improves charge separation and increases active sites for photocatalytic reactions. Moreover, the use of locally available and inexpensive kaolin as a support material enhances the economic feasibility of the developed photocatalyst. Therefore, these findings demonstrate the strong potential of the developed photocatalyst for the efficient removal of MB from aqueous solutions.

**Table 3:** Comparison of the MB degradation efficiency of the 3-Ag-Cu/g-C_3_N_4_/kaolin NC with previous report catalysts

Photocatalyst	Catalyst	MB mg/L (mL)	Light source	Irradiation time (min)	Efficiency (%)	Reference
3-Ag-Cu/g-C_3_N_4_/kaolin NC	15 mg	10 (50)	Sunlight	120	98.95	Present work
ZnFe₂O₄/rGO	10 mg	20 (100)	Visible light	180	93	[75]
NiO/Ag/TiO_2_	80 mg	5 (80)	Visible light	60	93	[76]
g-C_3_N_4_/CuO	100 mg	5 (100)	Visible light	40	98	[77]
g-C_3_N_4_/Fe_3_O_4_	125 mg	20 (250)	Visible light	120	90	[78]

### Photocatalytic activity and mechanism

**Fig. 10:**
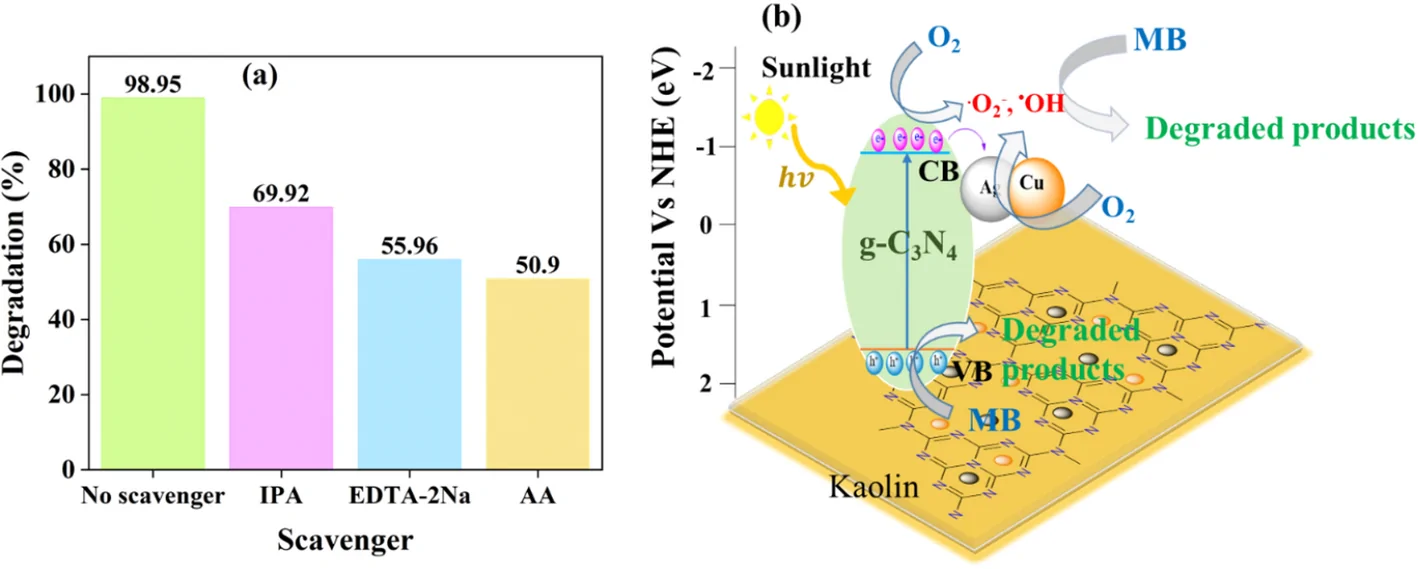
The effect of scavengers on the photocatalytic removal efficiency of MB (**a**), along with the proposed mechanism for the removal of MB using the 3-Ag-Cu/g-C₃N₄/kaolin NC under sunlight irradiation (**b**)

To elucidate the possible degradation mechanism and to identify the main reactive species during photocatalysis, various scavengers were utilized to quench the relevant active species. In this study, the MB dye solution was mixed with AA, IPA, and EDTA-2Na, which were utilized as quenchers of superoxide radicals (•O₂⁻), hydroxyl radicals (•OH), and holes (h^+^), respectively. As can be seen clearly from Fig. 10a, after 120 min of sunlight irradiation, the MB degradation efficiency decreased to 69.92% in the presence of IPA. Similarly, the degradation efficiency decreased to 55.96% and 50.9% upon the addition of EDTA-2Na and AA, respectively. The greater inhibition observed in the presence of EDTA-2Na and AA suggested that h⁺ and •O₂⁻ are the major reactive species contributing to MB degradation, whereas •OH plays a comparatively minor role.

Based on these results, a possible photocatalytic degradation mechanism is proposed. First, g-C₃N₄ absorbed the irradiated sunlight to generate photogenerated electron-hole pairs, and then the photoexcitation promoted electrons from the valence band (VB) of g-C₃N₄ to the conduction band (CB), leaving holes in the VB of g-C₃N₄. The presence of Ag-Cu NPs can further enhance visible-light utilization through the surface plasmon resonance (SPR) effect and facilitate interfacial charge separation [79]. The photogenerated electrons in the CB of g-C₃N₄ can migrate toward the surface of the Ag–Cu NPs, where they can react with dissolved molecular oxygen to generate superoxide radicals [80], which subsequently participate in photocatalytic removal of MB dye.

Meanwhile, the photogenerated holes (h^+^) remaining in the VB of g-C₃N₄ can directly oxidize adsorbed MB dye molecules [81]. The VB potential of g-C₃N₄ is generally not sufficiently positive to efficiently oxidize ^•^OH/H₂O and direct ^•^OH generation by VB holes of g-C₃N₄ is expected to be thermodynamically unfavorable. This observation is consistent with our results, where the photocatalytic removal efficiency showed a relatively small decrease upon the addition of IPA, indicating the comparatively small contribution of •OH radicals to MB photocatalytic removal. Nevertheless, the inhibition caused by IPA suggests that a certain amount of •OH may be generated through the multiple-electron reduction reaction of **·**O_2_ [79].

Overall, the scavenging experiments suggest that MB photocatalytic removal over the 3-Ag-Cu/g-C_3_N_4_/kaolin NC occurs through the combined action of photogenerated h^+^ and **·**O_2_ as the predominant reactive species, with •OH playing a comparatively minor role.

### Reusability of the photocatalyst

**Fig. 11:**
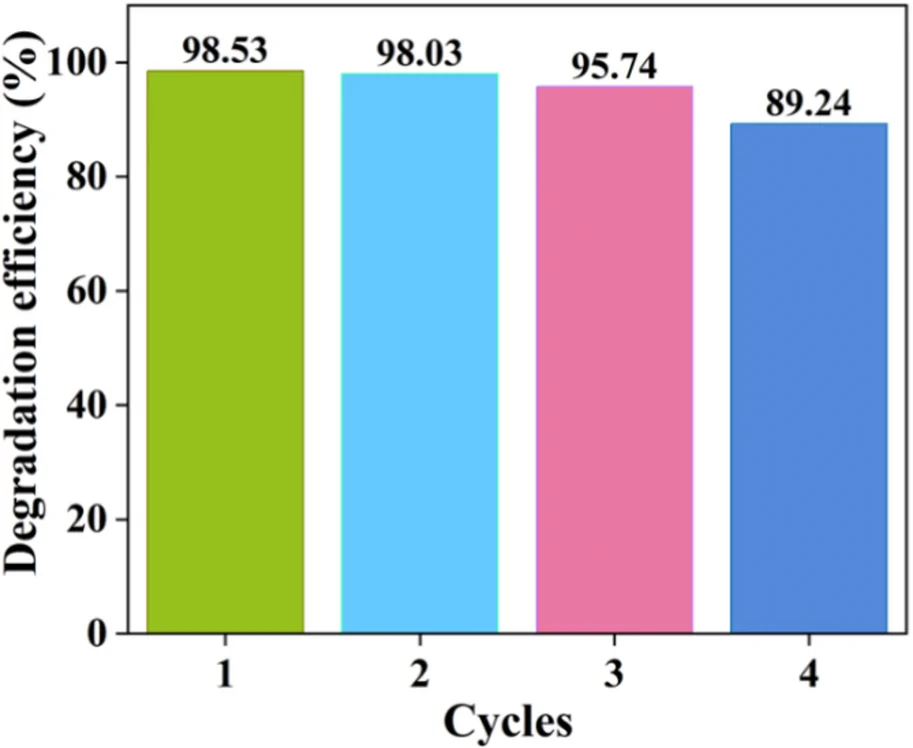
Stability tests of 3-Ag-Cu/g-C_3_N_4_/kaolin NC for the photocatalytic removal of MB dye under sunlight irradiation

From a practical application perspective, the stability and recyclability of photocatalysts are crucial for achieving sustainable wastewater treatment. Reusing these catalysts can lower treatment costs and mitigate the environmental impacts associated with catalyst disposal [82]. In order to examine the stability of the 3-Ag-Cu/g-C_3_N_4_/kaolin NC for MB removal, four cycles of tests were carried out under optimum conditions. As shown in Fig. 11, with four cycles of photocatalytic removal, no significant loss occurs. However, the minor decline in the percentage of photocatalytic removal observed can be the result of the partial deactivation of the catalyst following many reuses and some catalyst loss during centrifugation. Therefore, it can be concluded that the 3-Ag-Cu/g-C_3_N_4_/kaolin NC demonstrated good stability and recyclability during the photocatalytic removal processes of MB dye.

## Conclusions

This study presents a straightforward and economical approach for fabricating 3-Ag-Cu/g-C₃N₄/kaolin NC at ambient temperature, using a simple and affordable chemical reduction method. This NC was characterized using XRD, FT-IR, SEM/EDS, HRTEM, and XPS, and the combined characterization results provided evidence for the successful formation of the composite. This NC was employed for photocatalytic removal of MB dye from aqueous solution using natural sunlight and demonstrated superior performance, achieving a photocatalytic removal efficiency of 98.95% under optimized conditions. The enhanced photocatalytic efficiency of 3-Ag-Cu/g-C₃N₄/kaolin is attributed to the effective separation of electron-hole pairs due to the combination of g-C₃N₄ with Ag and Cu NPs.

Therefore, the results of this study demonstrated that the 3-Ag-Cu/g-C₃N₄/kaolin NC is an efficient visible light responsive photocatalytic material for the removal of MB and other toxic dyes from aqueous solutions. Due to its high dye removal efficiency and favorable reusability, this NC is anticipated to have strong potential for practical wastewater treatment applications.

Despite these promising results, this study has certain limitations that should be acknowledged. Individual-component control experiments were not conducted, and solar irradiance was not quantitatively monitored during the experiments. In addition, metal-ion leaching from the Ag–Cu/g-C₃N₄/kaolin NC was not quantified, although such analysis is important for evaluating catalyst stability and environmental safety. These aspects should therefore be addressed in future studies through systematic control experiments to determine the individual contributions of the catalyst components, quantitative irradiance measurements, and metal-leaching analysis.

## Data Availability

The datasets generated and/or analyzed during the current study are available from the corresponding author on reasonable request.
